# The role of men’s forgiveness in marital satisfaction and coping strategies of infertile iranian women

**DOI:** 10.1186/s12905-023-02389-x

**Published:** 2023-05-04

**Authors:** Samaneh Safari, Seyedeh Batool Hasanpoor-Azghady, Leila Amiri-Farahani

**Affiliations:** 1 Department of Midwifery and Reproductive, School of Nursing and Midwifery, Iran University of Medical Sciences, Rashid Yasemi st., Valiasr St, Tehran, 1996713883 Iran; 2 Department of Midwifery and Reproductive, Nursing and Midwifery Care Research Center, School of Nursing and Midwifery, Iran University of Medical Sciences, Tehran, Iran

**Keywords:** Forgiveness, Marital relationship, Coping strategies, Infertility

## Abstract

**Background:**

Infertility and its related problems create tensions in infertile women, which may lead to reduced marital satisfaction and the use of ineffective coping strategies. Considering the important role of forgiveness, marital satisfaction and effective coping strategies in the quality of life of infertile couples, and taking into account the growing number of Iranian infertile couples, this study was conducted to determine the relationship between men’s forgiveness, marital satisfaction, and coping strategies of infertile Iranian women.

**Methods:**

This cross-sectional study included 200 Iranian infertile couples. The research environment was the most equipped infertility center in the west of Iran. Sampling was continuous. Data collection tools used included a self-generated demographic and fertility questionnaire, the Family Forgiveness Scale (FFS), the Index of Marital Satisfaction (IMS), and the Ways of Coping Questionnaire-revised (WOCQ-R).

**Results:**

Husbands’ forgiveness had a significant direct relationship with the marital satisfaction of infertile women (r = -0.27, p < 0.001). However, there was no significant correlation between Husbands’ forgiveness, emotion-focused, and problem-focused coping of infertile women. Among the subscales of forgiveness, only the subscale of recognition had inversely correlated with the emotional coping of infertile women.

**Conclusion:**

The results showed that the higher the forgiveness of husbands, the higher the marital satisfaction of infertile women. Also, with the increase of husbands’ forgiveness in the recognition subscale, the use of emotion-focused coping decreased in infertile women. Based on the results with empowering the husbands of infertile women with forgiveness skills, it is possible to take a step towards marital satisfaction and thus improve the quality of life of infertile women.

## Introduction

One of the expectations of everyone from marriage is the birth of a child and reproduction [1]. This expectation is more pronounced in traditional societies [2]. Therefore, having a child is one of the most fundamental issues for most couples [3]. According to the results of a national plan, the infertility rate in Iran is 20.2% (19.9% ​​in cities and 22% in rural areas). This amount is far from the global average of 12–15% [4].

Infertility and its treatment, as a crisis in life, affects understanding couples of each other needs and desires. Therefore, it affects their satisfaction with marital life [5–7]. Infertility has a stressful nature. It can decrease psychological well-being by creating tension and stress. The pressure resulting from infertility affects positive personal, social and marital relationships, one of the dimensions of psychological well-being and causes a psychological imbalance in couples and marital incompatibility [8, 9]. However, some studies have reported that infertility does not affect marital satisfaction [6, 10, 11]. Some other studies have even reported a positive effect of infertility on marital satisfaction and increased couple intimacy [12, 13]. Most studies have shown the adverse effects of infertility on marital satisfaction [5, 14–17]. These studies show that infertility leads to boredom [14], decreased intimacy, fear of ending the relationship and helplessness in couples [17]. On the other hand, the husbands of infertile women face a series of arduous therapeutic and psychological activities [18, 19]. They are problems with sexual desire and marital relationships, feelings of guilt, hopelessness, depression, decreased self-esteem and feelings of emptiness in life [5, 20].

Two significant aspects of mental health include positive emotions and life satisfaction related to forgiveness [21]. Forgiveness is a powerful tool that ends a broken or painful relationship and provides the conditions for reconciliation with the partner. Therefore, removing one of the partners from the circle of negative interaction increases marital satisfaction and reduces conflict. [22]. Couples sometimes hurt each other in life. If they do not forgive the mistakes of their spouses, fall into the circle of negative interactions, their aggression towards each other will increase, their positive interactions will decrease, and they will experience less marital satisfaction [23]. The findings of a study showed a significant negative relationship between forgiveness and marital conflict [24]. Another study also showed that forgiveness is a predictor of marital satisfaction [25].

Forgiveness in couples is associated with emotion-focused coping, which is higher in women than men [26]. Coping responses are conscious efforts to control or reduce stress and acquisition to tolerate the threat that leads to stress [27]. Therefore, learning the necessary skills to use appropriate strategies is essential to reducing the problems caused by infertility [28]. Infertility problems cause negative thoughts in infertile people. The emotion-focused coping engages the person more with these negative thoughts. Negative thoughts constantly occupy a person’s mind and prepare him/her for depression. Meanwhile, in non-critical situations where the person does not necessarily experience negative emotions; emotion-focused coping may the positive function [13]. A quantitative study on infertile Iranian couples showed that couples use few effective coping methods like focusing on emotion and expressing it, negative thinking and wishful thinking, and methods such as denial and distancing. But, infertile couples, who are more determined in performing religious duties, use religious coping such as prayer, trust, and Patience [29].

Forgiveness is related to with adaptive coping strategies, focus on planning, positive reappraisal, perspective review and acceptance [30, 31]. Couples react to certain behaviors during marital conflicts and dissatisfaction with the situations that arise in conjugal relationships. Usually, these behaviors become a dominant behavioral pattern throughout their life. From this perspective, the frequency of behavioral patterns depends on the severity of the problem and conflict. In other words, the more intense and essential the issue for couples, the more tangible and recognizable behavioral patterns and coping strategies become [32]. Scientific evidence showed that strategies are the most important predictors of marital satisfaction. Strategies, especially coping strategies that control stress, can help increase the marital satisfaction of infertile women. [33]. For couples experiencing infertility, applying appropriate coping strategies has a positive effect on mitigating the stress caused by infertility and its treatment process [34, 35]. One study showed that, in women, forgiveness has positively correlated with emotions and acceptance coping strategies and is negatively related to negative evaluation and avoidance [26]. Infertility with psychosocial consequences in various forms requires strategies to reduce stress. These strategies can improve the quality of life of infertile people [36]. According to the evidence, most of the studies that examined the relationships between the variables of forgiveness, marital satisfaction and coping strategies, their research community consists of fertile individuals or couples. For this reason, this study was conducted with the aim of determining the relationship between men’s forgiveness, marital satisfaction, and coping strategies of infertile Iranian women.

## Methods

The present cross-sectional study included 200 infertile couples referring to the Infertility Treatment and Research Center of Omid Royan in the Central Province of Iran. Central Province has 32 cities and 66 villages. Center of Omid Royan is the most equipped infertility center in western Iran. The average number of visitors to this center is about 12,000 per year. About 80% of the clients are from Central Province and 20% from neighboring provinces. We selected eligible samples by continuous method.

To determine the sample size, we could not use studies that examined the relationship between forgiveness and marital satisfaction; because they had a high Pearson correlation coefficient and a small sample size was obtained. On the other hand, we did not find any study that examined the relationship between forgiveness and coping strategies. Therefore, r = 0.2 was used to determine the sample size, which means that two variables are correlated when they have at least 4% common variance [37]. We obtained a sample size of 200 couples with a confidence interval of 95%, test power of 80% and Pearson correlation coefficient of 0.2 between forgiveness, marital satisfaction and coping strategies.

The inclusion criteria were; having at least literacy to complete the questionnaires, having primary or secondary infertility with only female factor approved by the obstetrician, having no living child from secondary infertility, having a minimum of 1-year infertility treatment, having no adopted children, lack of other medical illnesses unrelated to infertility, absence of any mental illnesses requiring treatment based on self-report, not using any drugs, being in the first marriage in each couple, and lack of any tension-generating in the last six months such as loss of loved one, etc.

The data collection tools included a self-generated demographic and fertility questionnaire, Family Forgiveness Scale (FFS), Index of Marital Satisfaction (IMS), and Ways of Coping Questionnaire-revised (WOCQ-R).

## Family forgiveness scale (FFS)

FFS was designed and developed by Pollard et al. to measure forgiveness in families and couples, as well as the subscales of forgiveness. It has two sections. Each section has 20 items. The first section focuses on the family origin and the second section focuses on the primal relationship (nuclear family). Since the infertile couples in our study were childless, we used only the second section of the questionnaire (primal relationship). It has five subscales of realization, recognition, reparation, restitution, and resolution. Each subscale has four items, which are scored based on a four-point Likert scale, ranging from “never true (score = 1) to always true (score = 4)”. The score range of each subscale is between 4 and 16 and the score range of the entire scale is between 20 and 80, with the higher scores indicating more forgiveness. Pollard et al. obtained Cronbach’s alpha coefficient of 0.55–0.86 for the subscales of the FFS [38]. FFS has been psychometrically measured for the Iranian population by Seyf et al. in a study conducted on a sample of 766 married couples in Tehran (Iran). They reported the reliability of the second section of the scale at 85% based on Cronbach’s alpha [39].

## Index of marital satisfaction (IMS)

Hudson designed IMS to measure the degree, severity, or magnitude of the problem a spouse or partner has in a partner relationship. This scale consists of 25 items, of which 13 items are positively worded and 12 negatively worded. The positively worded items include 1, 3, 5, 8, 9, 11, 13, 16, 17, 19, 20, 21, and 23. The items IMS rate on a 7-point Likert scale ranging from 1 (None of the time) to 7 (All of the time). The overall score is obtained by summing up the 13 reverse-scoring items and adding them to the remaining scores. Then, the total number of completed responses given by the participant is subtracted, then the obtained figure is multiplied by 100. The score range of this scale is between 0 and 100. This scale has two clinical cutoff scores, the first of which is 30. Clients, who obtain a score of below 30, can be presumed not to have a clinically significant problem in this area. Clients, who obtain a score of above 30, can be presumed to have a clinically significant problem in this area. The second cutoff score is 70. Clients who obtain a score of 70 or higher are almost always experiencing severe distress. When distress reaches this level, there is a clear possibility that some form of violence will occur. This scale does not contain items about children that are suitable for our study in this regard. In the Hudson study, the IMS had test-retest reliability of 0.96 [40]. In Iran, Pouurakbar calculated the reliability of the IMS by test-retest at a distance of 15 days at 0.96. Also, for a more accurate evaluation, the reliability of the IMS was calculated at 0.88 using the split-half method [41].

## Ways of coping questionnaire-revised (WOCQ-R)

WOCQ-R was designed and revised by Lazarus and Folkman. It contains a wide range of coping and behavioral strategies people use to manage internal and external demands in stressful situations. The WOCQ-R consists of 66 items and eight subscales. The 16 items of this questionnaire are deviant phrases. This questionnaire measures two problem-focused and emotion-focused coping strategies. The problem-focused coping strategy of this questionnaire consists of four subscales, including accepting responsibility, seeking social support, Planful problem solving and positive reappraisal. The emotion-focused coping strategy of this questionnaire includes four subscales of confrontative coping, escape/avoidance, self-controlling and distancing. The items in this questionnaire rate on a 4-point Likert scale ranging from “does not apply and/or not used (score 0), used somewhat (score 1), used quite a bit (score 2), and used a great deal (score 3)”. WOCQ-R has two ways of scoring, raw and relative. The choice of scoring method depends on the information we are looking for it. Raw scores describe the coping effort for each of the eight types of coping. Relative scores describe the proportion of effort represented for each coping. It expresses as a percentage that ranges from 0 to 100. We used raw scoring in this study. In this way, the total score of each subscale is divided by the number of items in that subscale. Thus, the range of scores in each subscale is between 0 and 3. A high score indicates that the person has often used the behaviors described by that scale in coping with stressful events. Folkman et al. obtained Cronbach’s alpha coefficient of 0.61–0.79 for the subscales of this questionnaire [42]. In Iran, Nazarpour and Khazai reported a Cronbach’s alpha value of 0.59–0.79 for the subscales of this questionnaire [43].

Sampling began after approval of the project by the ethics committee of the Iran University of Medical Sciences with the code (IR.IUMS.REC.1397.544). After explaining the objectives of the study and the principle of confidentiality, the researcher obtained informed written consent from the eligible subjects. The study population of the present study were infertile couples due to female infertility. Husbands only completed the FFS, and infertile women completed the demographic and fertility questionnaire, IMS, and WOCQ-R. They completed the questionnaire by self-administered. Data were analyzed by SPSS software version 22 using independent t-test, one-way ANOVA, Kruskal-Wallis, and Pearson correlation tests. The significance level for all tests was p < 0.05.

## Results

Infertile women had a mean [± SD] age of 28.38 [± 4.63] years. The mean [± SD] age of the infertile women’s husbands was 33.08 [± 4.56] years. Couples had a mean [± SD] marriage duration of 6.9 [± 3.44] years. The mean [± SD] infertility duration of infertile women was 4.04 [± 2.40] years with a range of 1–13 years, and the mean [± SD] duration of infertility treatment was 2.73 [± 2.01] years with a range of 1–11 years. We presented more information about the demographic and fertility characteristics of the subjects in Table 1.

**Table 1:** Demographic and fertility characteristics in couples (n = 200)

Characteristics	N (%)	Characteristics	N (%)
Woman’s age (years)		Economic status	
< 25	47 (23.5)	Favorable	47 (23.5)
25–28	50 (25)	Relatively favorable	133 (66.5)
29–32	70 (35)	Undesirable	20 (10)
≥ 33	33 (16.5)	**Duration of the couple’s marriage (years)**	
**Spouse’s age (years)**		< 5	56 (28)
< 30	41 (20.5)	5–10	114 (57)
30–32	60 (30)	> 10	30 (15)
33–35	43 (21.5)	**Couples’ place of residence**	
≥ 36	56 (28)	City	185 (92.5)
**Women’s education**		Village	15 (7.5)
< High school	33 (16.5)	**Infertility duration (years)**	
High school	91 (45.5)	< 5	125 (62.5)
Academic	76 (38)	≥ 5	75 (37.5)
**Spouse’s education**		**Treatment Duration (years)**	
< High school	44 (22)	< 2	62 (31)
High school	86 (43)	2–4	110 (55)
Academic	70 (35)	5≤	28 (14)
**Woman’s occupation**		**Current treatment**	
Housewife	181 (90.5)	Drug	80 (40)
Employed	19 (9.5)	IUI	63 (31.5)
**Spouse’s occupation**		IVF	57 (28.5)
Employee	64 (32)	**Number of treatments**	
Free	104 (52)	1–2	125 (62.5)
manual worker	32 (16)	3–4	45 (27.5)
		5≤	30 (15)

Table 2 shows that the mean score of marital satisfaction was higher than 30. In other words, the infertile women had significant clinical problems. According to the cutoff points of the IMS, 110 (55%) of infertile women in the present study did not have a clinically significant problem, 78 (39%) of them had a clinically significant problem, and only 12 (6%) of them had severe stress and the possibility of domestic violence.

**Table 2:** Means and standard deviations of marital satisfaction and ways of coping of infertile women (n = 200)

Measure	mean	SD
**Marital satisfaction**	32.67	18.22
**Ways of coping**	**mean**	**SD**
Problem-focused coping	1.47	0.55
Emotional-focused coping	1.67	0.40

Table 3 showed that the fertility characteristics of couples had no statistically significant relationship with the forgiveness of husbands.

Table 4 shows the relationship between the forgiveness of husbands, marital satisfaction and coping strategies of infertile women.

**Table 3:** The relationship between forgiveness of husbands and fertility characteristics in couples (n = 200)

Characteristics		Forgiveness	
		Mean (SD)	P-value
^*^ **Infertility duration**	< 5	64.42 (6.13)	0.410
	≥ 5	63.68 (6.21)	
^**^ **Treatment Duration**	< 2	63.72 (6.40)	0.674
	2–4	64.81 (5.72)	
	5≤	64.46 (6.11)	
^***^ **Current treatment**	Drug	63.53 (5.74)	0.522
	IUI	64.49 (6.39)	
	IVF	64.61 (6.49)	
^**^ **Number of treatments**	1–2	64.40 (6.14)	0.345
	3–4	62.70 (6.19)	
	≤ 5	63.52 (6.28)	

^*^Independent t-test ^**^Kruskal–Wallis^***^One way ANOVA

**Table 4:** The Correlation between forgiveness, marital satisfaction, and ways of coping (n = 200)

Subscales of forgiveness in husbands	Marital satisfaction of infertile women		Problem-focused coping of infertile women		Emotional-focused coping of infertile women	
	r	^*^P-value	r	P-value^*^	r	^*^P-value
Realization	-0.16	0.021	-0.03	0.593	-0.12	0.091
Recognition	0.14	0.042	-0.07	0.277	-0.15	**0.033**
Reparation	-0.24	0.001	-0.085	0.230	-0.067	0.344
Restitution	-0.19	0.005	0.04	0.537	0.12	0.087
Resolution	-0.29	< 0.001	-0.12	0.074	0.02	0.760
Total FFS score	-0.27	< 0.001	-0.091	0.202	-0.05	0.475

^*^Pearson correlation tests

## Discussion

The results of this study, which aimed at determining the relationship between men’s forgiveness, marital satisfaction and coping strategies of infertile Iranian women, showed that with the increasing forgiveness of husbands, marital satisfaction of infertile women increases. However, the forgiveness of husbands did not have a significant relationship with the coping strategies of infertile women. According to the searches conducted by the researchers, no study was found that examines the relationship between forgiveness and marital satisfaction in the society of infertile women. Therefore, the researchers used studies with the research community of fertile people to compare the results.

The results of a study aimed at investigating attachment with marital satisfaction mediated by forgiveness and empathy in Iranian student couples showed that forgiveness and empathy, directly and indirectly, increase marital satisfaction [44], which was in line with the results of the present study. Findings of another study conducted on 1,513 married people in the United States showed a significant relationship between well-being, forgiveness and marital satisfaction [45]. Afkhami et al. reported that marital conflict significantly inversely correlated with the subscales of forgiveness including reparation, restitution recognition and realization [24]. This finding is in line with the results of the present study. Findings a study in Iran showed a significant relationship between the four subscales of forgiveness including realization, reparation, restitution, and recognition, and marital satisfaction of couples, but there was no significant relationship between the subscale of recognition and marital satisfaction [46].

Fahimdanesh et al. investigated the relationship between forgiveness and marital satisfaction in 200 Iranian couples with a marriage duration of one to ten years. The results showed that forgiveness predicts marital satisfaction in all samples of men and women. They suggested that family counselors should encourage couples to learn the skill of forgiveness and thereby improve their marital relationships [47]. Among other studies that had similar results to our findings, we can point to the study of Gaur and Bhardwaj aimed at examining relationship between empathy, forgiveness and marital adjustment in couples. The results of this study showed that marital compatibility had a direct relationship with high levels of forgiveness. The ability to forgive a life partner and willingness to forgive is one of the most important factors of marital compatibility [48]. Fincham et al. showed that forgiveness in couples predicts quality of care for each other, quality of communication, aggression and marital satisfaction [23]. A review article that examined the factors related to marital satisfaction showed that forgiveness is one of the factors affecting marital satisfaction [49].

The interpretations that scientific evidence provides for the relationship between forgiveness and marital satisfaction are as follows: Forgiveness is one’s moral response to another’s injustice [50], it is a powerful way to end a broken or painful relationship and provides reconciliation for the wrongdoers, and also marital forgiveness eliminates couples’ negative interactions and brings them back together. Such cohesion increases marital satisfaction and facilitates logical efforts to resolve inevitable marital conflicts. As a result, couples can find their healthy functioning. This model also ensures their mental health [23].

In the present study, there was no significant relationship between the forgiveness of husbands and emotion-focused coping and problem-focused coping of infertile women. However, there was a weak and inverse relationship between the subscale of recognition and emotion-focused coping in infertile women, in other words, the greater the forgiveness of husbands in terms of recognition, the lower the emotion-focused coping of infertile women. Regarding the relationship between forgiveness and coping strategies, by conducting extensive research, we obtained a few studies to compare the results of the present study.

Gabe and Monaghan showed that forgiveness has a significant positive relationship with problem-focused coping [30], which is contrary to the findings of the present study, but in line with our results, a study reported that forgiveness in women has a positive relationship with emotion-focused coping [26].

Emotion-focused coping includes distancing, escape/avoidance, confrontive coping, and self-controlling. Maltby et al. conducted a study to examine the cognitive nature of forgiveness. They evaluated the use of coping strategies in the forgiveness process. This study showed an inverse correlation between forgiveness and avoidance and a direct relationship between acceptance and forgiveness [26]. Similar to the results of our study, Zargar et al. stated in their study when a person accepts something, or in other words, recognizes it, his or her emotional behaviors will decrease [51]. Perhaps this explains the inverse effect of husbands’ recognition on the emotion-focused coping of their infertile wives in the present study. This reason can also be attributed to the relationship between the subscale of restitution and coping strategies because women gain more peace after appeasement [52]. It may be the reason why emotion-focused coping is reduced. It finds similar to the findings of the present study. In confirmation of this, Toussaint and Webb, which sought to determine the effect of gender differences on appeasement and forgiveness in Norwegian men and women, found that women had more appeasement than men, but restitution was associated more with forgiveness in men than in women [53].

### Study limitation

- Since the participants completed the questionnaires by self-reporting method, the response to some items might have been influenced by cultural factors and values ​​of the society in which the study samples live.

## Conclusion

The results showed that the higher the forgiveness of husbands, the higher the marital satisfaction of infertile women. Also, with the increase of husbands’ forgiveness in the recognition subscale, the use of emotion-focused coping decreased in infertile women. Based on the results, empowering the spouses of infertile women with forgiveness skills may lead to marital satisfaction and, as a result, the betterment of their quality of life.

## Data Availability

The datasets used and analyzed during the current study are available from the corresponding author on reasonable request.

## Abbreviations

FFS: Family Forgiveness Scale
IMS: Index of Marital Satisfaction
WOCQ-R: Ways of Coping Questionnaire-revised.
